# Tertiary Lymphoid Structures in Cancers: Prognostic Value, Regulation, and Manipulation for Therapeutic Intervention

**DOI:** 10.3389/fimmu.2016.00407

**Published:** 2016-10-03

**Authors:** Catherine Sautès-Fridman, Myriam Lawand, Nicolas A. Giraldo, Hélène Kaplon, Claire Germain, Wolf Herman Fridman, Marie-Caroline Dieu-Nosjean

**Affiliations:** ^1^INSERM, UMR_S 1138, Team “Cancer, Immune Control and Escape”, Cordeliers Research Center, Paris, France; ^2^UMR_S 1138, Centre de Recherche des Cordeliers, University Paris Descartes, Paris, France; ^3^UMR_S 1138, Centre de Recherche des Cordeliers, Sorbonne University, UPMC University Paris 06, Paris, France

**Keywords:** cancer, tertiary lymphoid structure, tumor microenvironment, chemokine, adaptive immune response

## Abstract

Tertiary lymphoid structures (TLS) are ectopic lymphoid aggregates that reflect lymphoid neogenesis occurring in tissues at sites of inflammation. They are detected in tumors where they orchestrate local and systemic anti-tumor responses. A correlation has been found between high densities of TLS and prolonged patient’s survival in more than 10 different types of cancer. TLS can be regulated by the same set of chemokines and cytokines that orchestrate lymphoid organogenesis and by regulatory T cells. Thus, TLS offer a series of putative new targets that could be used to develop therapies aiming to increase the anti-tumor immune response.

## Introduction

Tertiary lymphoid structures (TLS) are transient ectopic lymphoid organizations that develop after birth in non-lymphoid tissues, in situations of chronic inflammation. They display an overall organization similar to that observed in canonical secondary lymphoid organs (SLOs), such as lymph nodes (LNs), with a T cell-rich area characterized by a T cell and mature DC-Lamp^+^ dendritic cell (DCs) cluster, a B-cell-rich area composed of a mantle of naïve B cells surrounding an active germinal center (GC) (1–3), the presence of high endothelial venules (HEVs), a particular type of blood vessels expressing peripheral node addressins (PNAd) and specialized in the extravasation of circulating immune cells, and the secretion of chemokines (CCL19, CCL21, CXCL10, CXCL12, and CXCL13) that are crucial for lymphocyte recruitment and entry into the LN (4–8). TLS have been detected in the tumor invasive margin and/or in the stroma of most cancers and their densities correlate with a favorable clinical outcome for the patients (Table 1). A series of studies performed by our group in non-small-cell lung cancer (NSCLC) demonstrated that TLS are important sites for the initiation and/or maintenance of the local and systemic T- and B-cell responses against tumors, in accordance with a specific signature of genes related to T and B cell lineage, chemotaxis, Th1 polarization, lymphocyte activation, and effector function associated with TLS presence (Table 2). They represent a privileged area for the recruitment of lymphocytes into tumors and the generation of central-memory T and B cells that circulate and limit cancer progression (5, 9, 10).

**Table 1:** **Prognostic value of TLS in primary and metastatic tumors**.

Criteria	Cancer type	Stages of the disease	No. of patients	TLS detection IHC	TLS detection gene expression	Prognostic value	Reference
Primary tumors	Breast carcinoma	I–III	146	PNAd	–	Positive	(8)
		I–III	146	DC-Lamp	–	Positive	(11)
		I–III	794	–	T_FH_, CXCL13	Positive	(12)
	Breast carcinoma (triple negative)	I–III	769	H&S	–	Positive	(13)
	Colorectal cancer	I–IV	350	H&S	–	Positive	(14)
		ND	25	DC-Lamp	–	Positive	(15)
		I–IV	40	CD3, CD83	–	Positive	(16)
		II	185	CD3	–	Positive	(17)
		III	166	CD3	–	No value	(17)
		0–IV-A	21	–	12-chemokine genes	Positive	(3)
		I–IV	125	–	CXCL13 and CD20	Positive	(18)
	Gastric cancer	All without chemo	82	CD20	–	Positive	(19)
		I–III	365	–	both Th1 and B	Positive	(19)
	NSCLC	I–II	74	DC-Lamp	–	Positive	(1)
		I–IV	362	DC-Lamp	–	Positive	(9)
		III with neo-adj. chemo	122	DC-Lamp, CD20	–	Positive	(2)
	Melanoma	I-A–III-A	82	DC-Lamp	–	Positive	(20)
		IV	21	–	12-chemokine genes	Positive	(21)
	Oral SCC	All	80	CD3, CD20, CD21, BCL6, PNAd	–	Positive	(22)
	Pancreatic cancer	All	308 + 226	H&E	–	Positive	(23)
	RCC	All	135	DC-Lamp	–	Positive	(24)
	Hepatocellular Cancer	All	82	H&S	11-chemokine genes	Negative	(25)
	Biliary tract cancer	All	335	CD20 (TMA)	–	No value	(26)
Metastatic tumors	Colorectal cancer (liver)	All	14 + 51	CD20	–	Positive	(27)
	Colorectal cancer (lung)	ND	140	DC-Lamp	–	Positive	(15)

**Table 2:** **Expression of genes associated with TLS presence in human cancers**.

Name of the gene	Main names of the protein	Main immune functions and process	Cluster of gene related to TLS presence	Reference
CCL2	CCL2, MCP-1, MCAF	Monocyte, immature DC and T cell chemotaxis, G-protein-coupled receptor signaling pathway, cell adhesion, JAK-STAT cascade, MAPK cascade, cellular calcium ion homeostasis, cellular response to IFN-γ, IL-1, and IL-6	Chemotaxis	(3)
CCL3	CCL3, MIP-1α	Monocyte and T cell chemotaxis, G-protein-coupled receptor signaling pathway, cell adhesion, MAPK cascade, calcium-mediated signaling, cell activation, cellular response to IFN-γ, TNF-α, and IL-1, eosinophil degranulation, inflammatory response	Chemotaxis	(3)
CCL4	CCL4, MIP-1β, LAG1	Monocyte and neutrophil and T cell chemotaxis, G-protein-coupled receptor signaling pathway, cell adhesion, calcium-mediated signaling, cell activation, cellular response to IFN-γ, TNF-α, and IL-1, inflammatory response, positive regulation of ERK1 and ERK2 cascade, positive regulation of GTPase activity	Chemotaxis	(3)
CCL5	CCL5, RANTES	Monocyte, neutrophil and T cell chemotaxis, G-protein-coupled receptor signaling pathway, calcium-mediated signaling, cellular response to IFN-γ, TNF-α, and IL-1, inflammatory response	Chemotaxis	(3, 9)
CCL8	CCL8, MCP-2, HC14	Monocyte, neutrophil and T cell chemotaxis, G-protein-coupled receptor signaling pathway, cellular response to IFN-γ, TNF-α, and IL-1, chronic inflammatory response, positive regulation of ERK1 and ERK2 cascade, positive regulation of GTPase activity, negative regulation of leukocyte proliferation	Chemotaxis	(3)
CCL17	CCL17, TARC, ABCD-2	Monocyte and T cell chemotaxis, G-protein-coupled receptor signaling pathway, cellular response to IFN-γ, TNF-α, and IL-1, inflammatory response, positive regulation of ERK1 and ERK2 cascade, positive regulation of GTPase activity	Chemotaxis/T cells	(5)
CCL18	CCL18, PARC, MIP-4, AMAC-1, DC-CK1	Monocyte, neutrophil and T cell chemotaxis, G-protein-coupled receptor signaling pathway, cellular response to IFN-γ, TNF-α, and IL-1, inflammatory response, positive regulation of ERK1 and ERK2 cascade, positive regulation of GTPase activity	Chemotaxis	(3)
CCL19	CCL19, MIP-3β, ELC	Mature DC and T cell chemotaxis, G-protein-coupled receptor signaling pathway, T cell costimulation, cell maturation, cellular response to IFN-γ, TNF-α, and IL-1, inflammatory response, activation of JUN kinase activity, establishment of T cell polarity, immunological synapse formation, inflammatory response, positive regulation of IL-1β, IL-12, and TNF-α secretion, positive regulation of ERK1 ERK2 JNK cascade, response to PGE	Chemotaxis, chemotaxis/T cells	(3, 5)
CCL20	MIP-3α, LARC, Exodus	Immature DC monocyte neutrophil and T cell chemotaxis, G-protein-coupled receptor signaling pathway, cellular response to IL-1, TNF-α, and LPS, inflammatory response, positive regulation of ERK1 and ERK2 cascade	Th1/B cells	(19)
CCL21	CCL21, SLC, 6Ckine, TCA4	Mature DC neutrophil and T cell chemotaxis, G-protein-coupled receptor signaling pathway, T cell costimulation, cell maturation, cellular response to IFN-γ, TNF-α, and IL-1, inflammatory response, cell maturation, establishment of T cell polarity, negative regulation of DC dendrite assembly, positive regulation of DC APC function, immunological synapse formation, inflammatory response, activation of GTPase activity, cellular response to IL-1 and TNF-α, positive regulation of ERK1 ERK2 JNK cascade, response to PGE	Chemotaxis, chemotaxis/T cells	(3, 5)
CCL22	CCL22, MDC, ABCD-1, DC/B-CK	Monocyte and T cell chemotaxis, G-protein-coupled receptor signaling pathway, cellular response to IFN-γ, TNF-α, and IL-1, inflammatory response, positive regulation of ERK1 and ERK2 cascade, positive regulation of GTPase activity	Chemotaxis/T cells	(5)
CCR2	CCR2, CD192, CC-CKR2	Monocyte, immature DC and lymphocyte chemotaxis, G-protein-coupled receptor signaling pathway, positive regulation of inflammatory response, JAK–STAT cascade, negative regulation of eosinophil degranulation, positive regulation of Th1 immune response, negative regulation of Th2 immune response, positive regulation of IL-1β, IL-2, IL-6, and TNF production	Chemotaxis/Th1/cytotoxicity/activation	(9)
CCR4	CCR4, CD194, ChemR13, CC-CKR4	Monocyte and lymphocyte chemotaxis, G-protein-coupled receptor signaling pathway, inflammatory response, tolerance induction	Chemotaxis/Th1/cytotoxicity/activation	(9)
CCR5	CCR5, CD195	Myeloid and lymphocyte chemotaxis, G-protein-coupled receptor signaling pathway, inflammatory response, negative regulation of macrophage apoptotic process, positive regulation of IL-1, IL-6, and TNF production, co-receptor of HIV	Chemotaxis/Th1/cytotoxicity/activation, Th1/B cells	(9, 19)
CCR7	CCR7, CD197, CMKBR7, CC-CKR7, BLR2, EBI1	Monocyte mature DC and lymphocyte chemotaxis, G-protein-coupled receptor signaling pathway, inflammatory response, positive regulation of ERK1 and ERK2 cascade, positive regulation of GTPase activity, establishment of T cell polarity, negative thymic T cell selection, positive regulation of JNK cascade, positive regulation of T cell costimulation and TCR signaling pathway, positive regulation of APC function, positive regulation of humoral immunity, regulation of IFN-γ, IL-1β, and IL-12 production	Chemotaxis/Th1/cytotoxicity/activation	(9)
CD3e	CD3, TCRE, IMD18	T cell activation and costimulation, TCR signaling pathway, negative thymic T cell selection, positive regulation of T cell proliferation and anergy, positive regulation of IFN-γ, IL-2, and IL-4 production	Chemotaxis/T cells, chemotaxis/Th1/cytotoxicity/activation	(5, 9)
CD4	CD4	T cell activation, T cell differentiation, T cell selection, cytokine production	Chemotaxis/Th1/cytotoxicity/activation, Th1/B cells	(9, 19)
CD5	CD5, LEU1	T cell costimulation, apoptotic signaling pathway, cell proliferation, cell recognition, receptor-mediated endocytosis	Th1/B cells	(19)
CD8A	CD8A, Leu2, p32	T cell activation, T cell-mediated immunity, cell surface receptor signaling pathway, cytotoxic T cell differentiation, defense response to virus	Chemotaxis/Th1/cytotoxicity/activation	(9)
CD19	CD19, B4, CVID3	B-cell receptor signaling pathway, cell surface receptor signaling pathway, cellular defense response, phosphatidylinositol-mediated signaling, regulation of immune response	Chemotaxis/Th1/cytotoxicity/activation	(9)
CD20	CD20, MS4A1, LEU-16	B-cell lineage, B-cell proliferation, humoral immune response	Th1/B cells	(18, 19)
CD28	CD28, Tp44	T cell costimulation, TCR signaling pathway, negative thymic T cell selection, positive regulation of T cell proliferation, positive regulation of IL-2, IL-4, and IL-10 production, immunological synapse, positive regulation of isotype switching to IgG, humoral immune response	Chemotaxis/Th1/cytotoxicity/activation	(9)
CD38	CD38, ADPRC1	T cell activation, positive regulation of B-cell proliferation, B-cell receptor signaling pathway, negative regulation of apoptotic process, cell adhesion, calcium signaling, response to IL-1	Chemotaxis/Th1/cytotoxicity/activation, Th1/B cells	(9, 19)
CD40	CD40, TNFRSF5	B-cell proliferation, inflammatory response, positive regulation of B-cell proliferation, positive regulation of MAP kinase activity, positive regulation of IL-12 production, positive regulation of isotype switching to IgG, regulation of Ig secretion, TNF-mediated signaling pathway	Chemotaxis/Th1/cytotoxicity/activation, Th1/B cells	(9, 19)
CD40L	CD40 ligand, TRAP, CD154, HIGM1, TNFSF5, IGM	B-cell differentiation and proliferation, T cell costimulation, Ig secretion, isotype switching, negative regulation of apoptotic process, inflammatory response, positive regulation of NF-kappaB transcription factor activity, positive regulation of T cell proliferation, positive regulation of IL-4, IL-10, and IL-12 production, TNF-mediated signaling pathway	Chemotaxis/Th1/cytotoxicity/activation	(9)
CD62L	CD62L, L-selectin, LECAM1, LAM1	Cell adhesion, leukocyte migration, regulation of immune response	Chemotaxis/Th1/cytotoxicity/activation	(9)
CD68	CD68, LAMP4, GP110, SCARD1	Cellular response to organic substance	Chemotaxis/Th1/cytotoxicity/activation	(9)
CD80	CD80, B7, BB1, B7-1, CD28LG1	T cell activation, T cell costimulation, intracellular signal transduction, phosphatidylinositol-mediated signaling, positive regulation of Th1 cell differentiation, positive regulation of αβT cell proliferation, positive regulation of IL-2	Chemotaxis/Th1/cytotoxicity/activation	(9)
CD86	CD86, B7-2, B70, CD28LG2	B and T cell activation, T cell costimulation, cellular response to cytokine stimulus, DC activation, negative regulation of T cell anergy, phosphatidylinositol-mediated signaling, positive regulation of Th2 differentiation and T cell proliferation, positive regulation of IL-2 and IL-4 biosynthetic process, positive regulation of transcription and DNA-templated, response to IFN-γ, TLR3 signaling pathway	Chemotaxis/Th1/cytotoxicity/activation	(9)
CD200	CD200, OX-2	Regulation of immune response, negative regulation of macrophage activation, cell recognition	Tfh cells	(12)
CSF2	CSF2, GM-CSF	DC differentiation, macrophage activation, MAPK cascade, negative regulation of cytolysis, positive regulation of cell proliferation, positive regulation of IL-23 production, positive regulation of gene expression	Th1/B cells	(19)
CTLA-4	CTLA-4, CD152, IDDM12, ALPS5, GSE	T cell costimulation, negative regulation of Treg differentiation, negative regulator of B-cell proliferation, B-cell receptor signaling pathway, positive regulation of apoptotic process	Chemotaxis/Th1/cytotoxicity/activation	(9)
CXCL9	CXCL9, MIG, CMK	Neutrophil and T cell chemotaxis, Th1 polarization, G-protein-coupled receptor signaling pathway, inflammatory response, regulation of cell proliferation	Chemotaxis, Th1 orientation	(3)
CXCL10	CXCL10, IP10	Neutrophil monocyte and T cell chemotaxis, Th1 polarization, G-protein-coupled receptor signaling pathway, inflammatory response, positive regulation of cell proliferation	Chemotaxis, chemotaxis/Th1/cytotoxicity/activation	(3, 9)
CXCL11	CXCL11, IP9, I-TAC	T cell chemotaxis, Th1 polarization, G-protein-coupled receptor signaling pathway, inflammatory response, positive regulation of cell proliferation	Chemotaxis, chemotaxis/Th1/cytotoxicity/activation	(3, 9)
CXCL13	CXCL13, BLC, BCA1, SCYB13	B and Tfh cell chemotaxis, germinal center formation, lymph node development, regulation of humoral immunity, regulation of cell proliferation	Chemotaxis, chemotaxis/T cells, Tfh cells	(3, 5, 12, 18, 19)
CXCR3	CXCR3, CD182, CD183, GPR9	Neutrophil and T cell chemotaxis, Th1 polarization, G-protein-coupled receptor signaling pathway, inflammatory response, apoptotic process, cell adhesion, calcium-mediated signaling	Chemotaxis/Th1/cytotoxicity/activation	(9)
FasLG	Fas ligand, FASL, APTL, CD178, CD95L, TNFSF6, TNLG1A	T cell apoptotic process, activation of cysteine-type endopeptidase activity involved in apoptotic process, inflammatory cell apoptotic process, necroptotic signaling pathway, positive regulation of I-kappaB kinase/NK-kappaB signaling, positive regulation of cell proliferation, response to growth factor, transcription and DNA-templated	Chemotaxis/Th1/cytotoxicity/activation	(9)
FBLN7	FBLN7, Fibulin-7, TM14	Cell adhesion	Tfh cells	(12)
GF11	GF11	Regulation of transcription	Th1/B cells	(19)
GNLY	Granulysin, LAG2, NKG5	Cellular defense response, defense response to bacterium fungus, killing of cells of other organism	Chemotaxis/Th1/cytotoxicity/activation	(9)
HLA-DRA	HLA-DRA	T cell costimulation, TCR signaling pathway, antigen processing and presentation of exogenous peptide or polysaccharide antigen via MHC class II, IFN-γ-mediated signaling pathway, immune response	Chemotaxis/Th1/cytotoxicity/activation	(9)
ICAM-3	ICAM-3, CD50, ICAM-R	Cell adhesion, extracellular matrix organization, phagocytosis, regulation of immune response, stimulatory C-type lectin receptor signaling pathway	Chemotaxis/T cells	(5)
ICOS	ICOS, CD278	T cell costimulation, T cell tolerance induction, immune response	Chemotaxis/Th1/cytotoxicity/activation, Tfh cells	(9, 12)
IFN-γ	IFN-γ	T cell receptor signaling pathway, Th1-related cytokine	Chemotaxis/Th1/cytotoxicity/activation	(9)
IGSF6	IGSF6, DORA	Cell surface receptor signaling pathway, immune response	Th1/B cells	(19)
IL1R1	IL1RA, IL1R, CD121A	Cell surface receptor signaling pathway, IL-1-mediated signaling pathway, regulation of inflammatory response, response to TGF-β	Th1/B cells	(19)
IL1R2	IL1R2, CD121b, IL1RB	Inflammatory response, cytokine-mediated signaling pathway	Th1/B cells	(19)
IL-2	IL-2, lymphokine, TCGF	MAPK cascade, T cell differentiation, adaptive immune response, extrinsic apoptosis signaling pathway in absence of ligand, NK cell activation, negative regulation of B-cell apoptotic process, positive regulation of B and activated T cell proliferation, positive regulation of Ig secretion, positive regulation of IFN-γ and IL-17 production, positive regulation of isotype switching to IgG, positive regulation of Treg differentiation, regulation of T cell homeostatic proliferation	Chemotaxis/Th1/cytotoxicity/activation	(9)
IL2RA	IL2RA, CD25, IL2R, p55	Activation-induced cell death of T cells, positive regulation of activated T cell proliferation, positive regulation of T cell differentiation, inflammatory response, IL-2-mediated signaling pathway, regulation of T cell tolerance induction	Th1/B cells	(19)
IL-10	IL-10, TGIF, GVHDS, CSIF	B-cell differentiation, inflammatory response, negative regulation of T- and B-cell proliferation, negative regulation of apoptotic process, negative regulation of cytokine activity, negative regulation of IFN-γ, IL-1, IL-12, IL-18, IL-6, IL-8, and TNF production, negative regulation of myeloid DC activation, positive regulation of JAK-STAT cascade, regulation of isotype switching, Th3/Tr1/regulatory immune responses	Chemotaxis/Th1/cytotoxicity/activation, Th1/B cells	(9, 19)
IL-12B	IL12B, CLMF, NKSF, IMD28, IMD29	Positive regulation of Th1 and Th17 immune responses, Th differentiation, cellular response to IFN-γ, defense response to virus, positive regulation of NK and T cell activation, positive regulation of memory T cell differentiation, regulation of IL-10, IL-12, IL-17, TNF-α, and GM-CSF production, positive regulation of NK T cell activation and proliferation, positive regulation of T cell-mediated cytotoxicity, regulation of tyrosine phosphorylation of STAT1	Chemotaxis/Th1/cytotoxicity/activation	(9)
IL-15	IL-15	NK T cell proliferation, extra-thymic T cell selection, inflammatory response, LN development, positive regulation of NK and T cell proliferation, positive regulation of IL-17 production, signal transduction, tyrosine phosphorylation of STAT5	Chemotaxis/Th1/cytotoxicity/activation	(9)
IL-16	IL-16, LCF, NIL16	Immune response, induction of positive chemotaxis, regulation of transcription and DNA-templated	Chemotaxis/T cells	(5)
IL-18	IL-18, IGIF, IL1γ, IL1F4	MAPK cascade, Th1/Th2 immune response, GM-CSF biosynthetic process, inflammatory response, IFN-γ, IL-2, and IL-13 biosynthetic process, NK cell activation and proliferation, positive regulation of IL-17 and IFN-γ production, positive regulation of tyrosine phosphorylation of STAT3	Chemotaxis/Th1/cytotoxicity/activation	(9)
IRF4	IRF4, MUM1, LSIRF	T cell activation, Th17 cell lineage commitment, IFN-γ-mediated signaling pathway, positive regulation of IL-10, IL-13, IL-2, and IL-4 biosynthetic process, regulation of Th cell differentiation, Type-I IFN signaling pathway, positive regulation of transcription	Th1/B cells	(19)
ITGAL	ITGAL, CD11A, LFA-1	Extracellular matrix organization, T cell activation via TCR contact with antigen bound to MHC molecule on APC, leukocyte migration, heterotypic cell–cell adhesion, immune response, integrin-mediated signaling pathway, inflammatory response, phagocytosis, regulation of immune response	Chemotaxis/T cells	(5)
ITGAD	ITGAD, ADB2, CD11D	Extracellular matrix organization, heterotypic cell–cell adhesion, immune response, integrin-mediated signaling pathway	Chemotaxis/T cells	(5)
ITGA4	ITGA4, CD49D	B-cell differentiation, cell-matrix adhesion, diapedesis, extracellular matrix organization, heterotypic cell–cell adhesion, integrin-mediated signaling pathway, leukocyte migration tethering or rolling, regulation of immune response	Chemotaxis/T cells	(5)
LTA	LTA, Lymphotoxin α, TNFB, TNFSF1	Positive regulation of apoptotic process, cell–cell signaling, positive regulation of humoral immune response mediated by circulating Ig, LN development, positive regulation of IFN-γ production, TNF-mediated signaling pathway	Chemotaxis/Th1/cytotoxicity/activation	(9)
MADCAM1	MADCAM1	Cell–matrix adhesion, extracellular matrix organization, heterotypic cell–cell adhesion, integrin-mediated signaling pathway, leukocyte tethering or rolling, receptor clustering, regulation of immune response, signal transduction	Chemotaxis/T cells	(5)
PDCD1	PD-1	T cell costimulation, humoral immune response, positive regulator of T cell apoptotic process	Tfh cells	(12)
PRF1	Perforin, PFP, FLH2, PFN1	Apoptotic process, cellular defense response, cytolysis, defense response to tumor cell, immunological synapse formation, transmembrane transport	Chemotaxis/Th1/cytotoxicity/activation	(9)
SDC1	SDC, CD138, syndecan	Cell migration, inflammatory response, canonical Wnt signaling pathway	Th1/B cells	(19)
SGPP2	SGPP2, Spp2, SPPase2	Regulation of immune response, positive regulation of signal transduction, positive regulation of NK-mediated cytotoxicity	Tfh cells	(12)
SH2D1A		Signal transduction of T- and B-cell activation	Tfh cells	(12)
STAT5A	STAT5A, MGF	JAK–STAT cascade, peptidyl-tyrosine phosphorylation, regulator of transcription	Th1/B cells	(19)
TBX21	T-Bet, TBLYM	T cell differentiation, lymphocyte migration, positive regulation of transcription and DNA-templated, positive regulation of isotype switching to IgG	Chemotaxis/Th1/cytotoxicity/activation	(9)
TIGIT	TIGIT, VSTM3, VSIG9	T cell co-inhibitory receptor, negative regulation of IL-12 production, positive regulation of IL-10 production	Tfh cells	(12)
TNF-α	TNF-α, DIF, TNFSF2	I-kappaB kinase/NF-kappaB signaling, JNK cascade, MAPK cascade, activation of MAPK and MAPKKK activities, humoral immune response, inflammatory response, necroptotic signaling pathway, negative regulation of cytokine secretion, negative regulation of cytokine and chemokine production, negative regulation of transcription and DNA-templated, positive regulation of ERK1 and ERK2 cascade, positive regulation of I-kappaB kinase/NF-kappaB signaling, positive regulation of JUN and MAP kinase activity, positive regulation of apoptotic process, positive regulation of humoral response and Ig secretion	Chemotaxis/Th1/cytotoxicity/activation	(9)
TRAF6	TRAF6, RNF85	FcE receptor signaling pathway, JNK cascade, MyD88-dependent TLR signaling pathway, MyD88-independent TLR signaling pathway, TCR signaling pathway, Th1 immune response, activation of MAPK activity, Ag processing and presentation of exogenous peptide Ag, myeloid DC differentiation, positive regulation of T cell activation proliferation and cytokine production, positive regulation of IL-12 production, response to IL-1, TLR signaling pathway	Th1/B cells	(19)
VCAM-1	VCAM-1, CD106	B-cell differentiation, acute inflammatory response, cell–matrix adhesion, cellular response to TNF-α and VEGF, IFN-γ-mediated signaling pathway, leukocyte tethering or rolling, positive regulation of T cell proliferation, regulation of immune response, response to hypoxia	Chemotaxis/T cells	(5)

*Genes selectively overexpressed in tumors having high density of TLS in cancer patients*.

In this mini review, we summarize the available data in the literature regarding the prognostic value of TLS in human cancers, and discuss how these structures are controlled and could be manipulated in order to increase anti-tumor immune responses.

## TLS and Prognosis in Cancers

**Figure 1:**
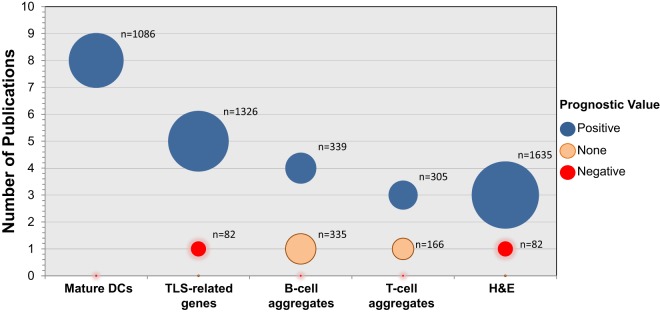
**Prognostic value of TLS-associated biomarkers in primary and metastatic cancers**. The number of publications studying the impact of mature DCs, TLS-related gene signatures, B-cell aggregates, T cell aggregates, or H&E with regard to prognosis in human cancers is represented (12 cancer types have been included). Blue, orange, and red circles represent an association with good, none, and poor prognosis, respectively. The diameter of the circles represents the total number of tumors (*n*) that have been analyzed on these studies.

In recent years, numerous publications have assessed the prognosis associated with the presence of TLS in different types of tumors. Several strategies for their quantification have been used. Historically, the first method to measure the densities of TLS was the quantification of mature DCs (DC-Lamp^+^) within CD3^+^ T cell aggregates (1, 20). Although relatively challenging due to the relative low number of DC-Lamp^+^ DCs in some tumors (as compared to other immune populations), our group has described it as the most accurate marker for quantifying TLS (28, 29). Up-to-date, eight publications have found a positive association between increased densities of DC-Lamp^+^ DCs and prognosis, in several types of tumors, including NSCLC (1, 2, 9), melanoma (20), renal cell carcinoma [RCC (24)], breast (11), and colorectal cancer (15) (Table 1 and Figure 1).

The analysis of expression levels of TLS-related genes gives the possibility to rapidly assess the prognostic impact of these immune aggregates in large retrospective cohorts of tumors. So far, six studies have evaluated the prognostic impact of increased expression of TLS-related genes in cancer. Despite heterogeneity in the TLS-signatures, a significant correlation with good prognosis has been found in melanoma (21), colorectal (3, 18), and gastric (19) cancers (Table 1). Interestingly, TLS found in inflammatory zones from hepatocellular carcinoma (HCC) correlate with increased risk for late recurrence and a trend toward decreased overall survival after HCC resection. This result could reflect an unexpected role for TLS, serving as niche for HCC progenitor cells *via* local production of Lymphotoxin (LT)-β (25, 30).

Another approach that has been used to estimate the densities of TLS in cancers is the quantification of B-cell aggregates by immunohistochemistry (IHC) (CD20^+^ B-cell aggregates or islets). The majority of publications measuring CD20^+^ aggregates (four out of five), accounting for more than 349 analyzed tumors, has determined that increased densities of this population correlate with good prognosis in several neoplasias, such as NSCLC (2), colorectal cancer liver metastasis (27), gastric (19), and oral (22) cancer (Table 1 and Figure 1). Most of the studies quantifying the CD3^+^ T cell aggregates and immune-cell aggregates (after hematoxylin counterstaining) have also found a positive impact on patient’s prognosis. However, high numbers of B cell or T cell aggregates were found to have no impact on prognosis in biliary tract cancer and in stage III colorectal cancer, respectively. Further studies are needed to investigate whether it reflects that cell aggregates counting is not an accurate method to quantify TLS, or a functional impairment of TLS in these two cancer types (Table 1 and Figure 1).

Overall, despite the heterogeneity of methods used for quantifying TLS, most of the studies have consistently found a correlation between high densities of TLS and prolonged patient’s survival in more than 10 different types of cancer (Table 1). Further efforts should be made to optimize TLS-quantifying methods. Indeed the use of multicolor IHC will facilitate their characterization, by allowing the simultaneous detection of all major cell types and providing an extensive analysis of their cellular complexity.

## TLS Neogenesis

The cellular composition and spatial organization of TLS share many similarities with those of SLO. Indeed, an increasing number of studies performed in a large variety of inflammatory disorders, in mice and in humans, suggest that their formation and regulation involve the same set of chemokines than those acting in lymphoid organogenesis.

### Positive Regulators

Lymphotoxin, CCL21, and CXCL13 were shown to play a major role during TLS neogenesis, and are related to TLS presence in human tumors (Table 2). In a mouse model of atherosclerosis, the activation of LTβR^+^ medial smooth muscle cells in the abdominal aorta by LT produced by CD11c^+^ CD68^+^ Ly6C^lo^ monocytes leads to the expression of CCL19, CCL21, CXCL13, and CXCL16 chemokines, which in turn trigger the recruitment of lymphocytes to the adventitia and the development of TLS (31). The same observation was made by Thaunat et al. in a rat model of chronic allograft rejection, in which M1-macrophages behaved as LTi cells in diseased arteries by expressing high levels of LTα and TNF-α (32). In human NSCLC, a TLS-related gene signature was identified, including CCL19, CCL21, IL-16, and CXCL13 (5) (Table 2). Interestingly, Matsuda et al. recently suggested in a mouse intrapulmonary tracheal transplant model that lymphoid neogenesis was dependent on spleen tyrosine kinase (Syk)-signaling. Decreased expression of CXCL12, CXCL13, and VEGF-α, lower B-cell recruitment into allograft, and smaller lymphoid aggregate area were observed in Syk-deficient recipient mice as compared to controls (33).

The generation of HEVs is also a critical step in TLS neogenesis. HEV endothelial cells express LTβR, and the continuous engagement of LTβR on HEVs by LT^+^ CD11c^+^ DCs is critical for the induction and maintenance of the mature HEV phenotype required for the extravasation of blood lymphocyte into LNs (34–37). In addition, CD11c^+^ DCs can be sources of proangiogenic factors, such as VEGF, favoring the development of HEVs, and ultimately lymphocyte entry into LN (38–41). Consistently, LTβ expression correlates with that of HEV-associated chemokines in human breast cancer, and DC-Lamp^+^ DC density correlates with HEV density, lymphocyte infiltration, and favorable clinical outcome (11). Other cell types were shown to favor the development of HEV. For instance, ectopic expression of CCL21 in the thyroid gives rise first to the recruitment of CD3^+^ CD4^+^ T cells followed by DC, and this DC-T cross-talk is required for the local development of both TLS and mature HEV (42). Tumor-infiltrating CD8^+^ T cells and NK cells were also shown to drive the *de novo* development of PNAd^+^ TNFRI^+^ CCL21^+^ HEV-like blood vessels through the production of LT and IFN-γ (43).

Th17 cells share many developmental and effector markers with LTi cells, including the nuclear hormone receptor retinoic acid-related orphan receptor γt (RORγt), which promotes not only the production of IL-17 and IL-22 by Th17 cells, LTi cells, and other RORγt^+^ innate lymphoid cells (ILCs), but also cell membrane expression of LT [reviewed in Ref. (44)]. In mice lungs, the formation of TLS [called here induced-bronchus-associated lymphoid tissues (i-BALT)] following LPS sensitization was dependent of IL-17 production by T cells, including Th17 and γδ T cells (45). This observation was also observed in a mouse experimental autoimmune encephalomyelitis (EAE) model of multiple sclerosis (46). Similarly, IL-17α-deficient mice exposed to cigarette smoke displayed decreased number of ectopic lymphoid follicles and decreased expression of CXCL12 as compared to wild-type mice in a model of chronic obstructive lung disease (47). It has also been suggested that Th17 cells, and IL-17 and IL-21 secretion by these cells can promote TLS neogenesis within human renal grafts, and are associated with the presence of active GC B cells and fast chronic rejection (48).

Other inflammatory cytokines also seem to promote TLS neogenesis. In rheumatoid arthritis (RA), high protein levels of IL-23 and IL-17F were detected in the synovial fluid of patients displaying ectopic lymphoid follicles, and a positive correlation was observed between CD21L mRNA (as a TLS marker) and IL-23 but also IL-17F, IL-21, and IL-22 mRNAs (49). IL-22 was also proposed to favor TLS induction (50). In a mouse model of virus-induced autoantibody formation in the salivary glands, it was shown that the ligation of IL-22R expressed by epithelial cells and fibroblasts leads to CXCL12 and CXCL13 production, allowing B-cell recruitment and TLS organization. In that case, IL-22 was mainly produced by γδ T cells and to a lesser extent by ILCs and NK cells during the early phase post-infection, and then by αβ T cells later after infection.

### Negative Regulators

On the opposite, IL-27, a cytokine known to inhibit effector Th17 responses was recently suggested to negatively regulate the development of ectopic lymphoid-like structures in the synovial tissues of RA patients. While patients having a high density of TLS displayed high synovial levels of IL-17 and IL-21, high levels of IL-27 were observed in patients devoid of any TLS, and IL-27 expression was inversely correlated with CD3^+^ and CD20^+^ infiltrates and with synovitis. This observation was confirmed in a mouse model of RA (51).

Among the immune cells infiltrating tumors are regulatory T cells (Tregs), which have been considered in many reports as a marker of poor prognosis in cancer (52, 53). Tregs have been reported to negatively interfere with BALT development. Indeed in CCR7-deficient mice, BALTs developed spontaneously in the absence of infection, an event that is directly reverted by the adoptive transfer of wild-type Tregs but not CCR7^−/−^ Tregs (54). In human breast cancer, Tregs were detected in lymphoid aggregates surrounding tumor nests, and their presence was linked with the poor clinical outcome of patients (55). In mice bearing breast tumors, Treg depletion led to an increased density of HEV within the tumor, facilitated T cell recruitment from the blood, and ultimately induced tumor destruction (56). This observation is in accordance with a human study showing that HEV^high^ breast tumors correlated with a high LT-β expression, a high density of tumor-infiltrating mature DC, and a decreased FoxP3^+^/CD3^+^ T cell ratio (11).

More recently, a new mechanism involving regulation of TLS formation by Tregs was found, by dampening neutrophilic inflammation (57). The presence of neutrophils seemed to be critical for the neogenesis and the humoral immune function of i-BALT by enhancing B-cell activation and survival, Ig class switching to IgA as well as plasma cell survival (57).

Regulatory T cells have been shown to dampen the effector T cell response promoted within tumor-associated TLS. Treg depletion causes immune-mediated tumor destruction associated with an increased expression of co-stimulatory ligands by DCs and proliferation of T cells in a murine model of lung adenocarcinoma (58). Further studies should be carried out to analyze the prognostic importance of Tregs and their immunosuppressive potential in cancer patients according to their localization.

Altogether, TLS neogenesis and lymphoid organogenesis share many common mechanisms. On the one hand, the production of inflammatory cytokines (LT, IL-17, IL-22, and IL-23) and lymphoid chemokines (CCL21, CXCL12, and CXCL13), HEV development as well as the activation of DCs, B, and effector cells seem to be crucial events leading to TLS neogenesis under inflammatory conditions, such as cancers. On the other hand, the presence of Tregs appears to negatively impact TLS formation and TLS-associated T cell responses.

## Manipulation of TLS for a Therapeutic Intervention in Cancer

A series of studies suggest that TLS are sites for generation and maintenance of adaptive anti-tumor responses (10). Therefore, TLS induction could be used as a therapeutic intervention for a better tumor control and prolonged survival of cancer patients. Since LN and TLS share many similarities in terms of cellular composition and organization, deciphering the mechanisms of lymphoid organogenesis enables to first highlight some putative key molecules that can support TLS neogenesis.

### Targeting Molecules Involved in Lymphoid Organogenesis

The key cross-talk between LTi cells and lymphoid tissue organizer cells (LTo cells that are cells of mesenchymal origin) occurring during LN development involves several molecules along with RANK and its ligand, which lead to LTβR signaling (59). Therefore, targeting RANK/LT pathway may modulate TLS development through the activation of LTo cells. Currently, antagonists of LTα (Pateclizumab NCT01225393), LTβR (Baminercept, NCT01552681) and RANK signaling (NCT01973569) are under investigation in several inflammatory situations. A special attention should be made in cancer setting where these antagonists might block TLS formation and, hence, reduce survival. The use of agonists might rather present a benefit to cancer patients but no drugs have been developed so far.

Activation of LTβR signaling pathway in LTo cells induces VCAM-1 and ICAM-1 upregulation, and ultimately leukocyte infiltration (60). Because both molecules are known to be induced by inflammation, an ICAM-1 antagonist called Alicaforsen has been tested in autoimmune diseases (NCT00048113, NCT00063830). We can speculate that the development of VCAM-1/ICAM-1 agonists would promote LTi-like cells-LTo clusterings and improve the leukocyte recruitment in order to generate cancer-associated TLS.

IL-7 receptor (CD127) signaling has been reported as a key pathway for TLS neogenesis (61). IL-7 is not only crucial for the survival and proliferation of LTi cells but also for GC formation and Tfh differentiation (62). To date, only one pharmacologic agent (IL-7R) is under investigation in NOD mice to deplete autoreactive T cells and to regulate pro-inflammatory mediators (63).

Altogether, as a counterpart of autoimmune diseases, development of agonist molecules targeting lymphoid organogenesis might be a promising strategy for the initiation and the maintenance of TLS in cancers.

### Modulation of Chemokine and Cytokine Networks

Lymphoid chemokines represent a good therapeutic target for the modulation of TLS (Table 2). The CCL19–CCL21/CCR7 and CXCL13/CXCR5 couples are induced after LT-βR signaling during lymphoid genesis (60). They are overexpressed in TLS of melanoma (21), colorectal (3), and lung (5) cancer patients. Using lymphoid chemokines or their agonists could be a promising strategy to induce TLS neogenesis in cancers. For example, CCL21 has been shown to attract circulating naïve T cells and DCs in tumors, and contribute to the control of tumor growth (64–66). A Phase I clinical trial is currently under investigation in NSCLC patients receiving intra-tumoral injections of CCL21-transduced autologous DCs (NCT00601094, NCT01574222). It is tempting to speculate that this vaccine therapy would boost TLS formation in tumors associated with an influx of lymphocytes, an effective anti-tumor immune response, and a reduction of tumor burden.

IL-21, which is mainly secreted by Th17 cells and neutrophils, represents also an interesting molecular target. First, this cytokine has been shown to promote TLS neogenesis in lungs after acute LPS exposure and IL-21^−/−^ mice exhibit fewer TLS in allografts than the control group (57). Second, IL-21 can enhance B and plasma cell survival as well as B-cell-dependent immunity, and induce conventional T cells to become refractory to Treg immunosuppression (48, 57, 67). Even if IL-21 can block IL-2 production with deleterious consequences in terms of Treg differentiation, IL-21 can substitute for IL-2 as a T cell growth factor (68). Recombinant IL-21 is currently tested in many clinical trials, alone or in combination with chemotherapy, therapeutic antibodies or tyrosine kinase inhibitors (e.g., NCT00617253, NCT00389285, NCT00095108, NCT01629758, NCT00336986, and NCT01489059). Altogether, it is likely that IL-21 could promote a robust anti-tumor immunity in a TLS-dependent manner.

## Conclusion and Perspectives

By facilitating the direct entry of CCR7^+^ naïve T cells and CXCR5^+^ B cells into tumors through HEVs, TLS allow T cells to differentiate into effector cells upon contact with mature DCs and B cells to form GC, protected from the immunosuppressive milieu of the tumor microenvironment. Therefore, TLS represent sites for the induction and maintenance of the local and systemic anti-tumor responses, which confer long-term protection against metastasis and, hence, correlate with good prognosis for the patients. Indeed, therapies aiming to increase TLS formation may allow generating anti-tumor responses directly *in situ* and would be beneficial in patients with high mutational load. TLS may also constitute biomarkers of anti-tumor response in patients undergoing immunotherapies. Thus, TLS induction was observed in cervical cancer patients vaccinated with HPV DNA (69) or with G-VAX (70), and one may speculate that TLS signature could be used to evidence response to therapies that unlock the adaptive immune responses.

## Author Contributions

ML, NG, HK, CG, and CSF wrote and revised the paper. WF and MCDN revised the paper.

## Conflict of Interest Statement

The authors declare that the research was conducted in the absence of any commercial or financial relationships that could be construed as a potential conflict of interest.
